# The effects of myosteatosis on skeletal muscle function in older adults

**DOI:** 10.14814/phy2.16042

**Published:** 2024-05-05

**Authors:** Kathleen Dondero, Ben Friedman, Julie Rekant, Rian Landers‐Ramos, Odessa Addison

**Affiliations:** ^1^ Department of Physical Therapy and Rehabilitation Science University of Maryland School of Medicine Baltimore Maryland USA; ^2^ Department of Kinesiology Towson University Towson Maryland USA; ^3^ Baltimore Geriatric Research, Education, and Clinical Center Baltimore Veterans Affairs Medical Center Baltimore Maryland USA

**Keywords:** aging, muscle function, muscle stiffness, myosteatosis

## Abstract

Myosteatosis, or the infiltration of fatty deposits into skeletal muscle, occurs with advancing age and contributes to the health and functional decline of older adults. Myosteatosis and its inflammatory milieu play a larger role in adipose tissue dysfunction, muscle tissue dysfunction, and increased passive muscle stiffness. Combined with the age‐related decline of sex hormones and development of anabolic resistance, myosteatosis also contributes to insulin resistance, impaired muscle mechanics, loss of force production from the muscle, and increased risk of chronic disease. Due to its highly inflammatory secretome and the downstream negative effects on muscle metabolism and mechanics, myosteatosis has become an area of interest for aging researchers and clinicians. Thus far, myosteatosis treatments have had limited success, as many lack the potency to completely rescue the metabolic and physical consequences of myosteatosis. Future research is encouraged for the development of reliable assessment methods for myosteatosis, as well as the continued exploration of pharmacological, nutritional, and exercise‐related interventions that may lead to the success in attenuating myosteatosis and its clinical consequences within the aging population.

## Graphical abstract

Muscle dysfunction and adipose tissue dysfunction contribute to passive muscle stiffness, and the decline of muscle protein synthesis promotes a catabolic state—encouraging the infiltration of adipose tissue and resulting in the loss of force production.

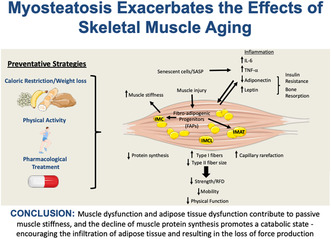

## 1 INTRODUCTION

Myosteatosis, the infiltration of fat into skeletal muscle, is an umbrella term describing the deposition of adipose into various regions of skeletal muscle. Myosteatosis is clinically relevant due to its role as a risk factor in mobility limitations (Correa‐de‐Araujo et al., 2020; Justice et al., 2018), morbidity (Addison, Marcus, et al., 2014), and mortality (Rossi et al., 2021). The term encompasses (a) intermuscular adipose tissue (IMAT), the extracellular fat deposits between a muscle and its overlying fascia and neighboring muscles, (b) intramuscular adipose tissue (IMC), found between muscle cells within an individual muscle, and (c) intramyocellular lipids (IMCL), found within muscle cells themselves (Correa‐de‐Araujo et al., 2020). With advancing age, pathologies, and decreased activity levels, older adults experience a redistribution of adipose tissue, reducing subcutaneous fat and increasing IMAT and IMC depots (Waters, 2019). The relationship between IMCL and muscle is complex. The phenomenon of the athlete's paradox in which IMCLs are increased in both athletes and obese individuals is a noted example of this complexity (Li et al., 2019). While a full review of the cellular origin and pathways of fat infiltration is beyond the scope of this paper, it has been recently extensively reviewed elsewhere (Wang et al., 2024). The opportunity for ectopic fat depots to infiltrate dysfunctional muscle tissue (Goodpaster & Wolf, 2004) contributes to the development of myosteatosis, though it appears to be more complex than just the mere replacement of muscle fibers with adipose tissue. For instance, Ilich et al. found that the endocrine interplay (such as leptin and adiponectin) between neighboring adipose and muscle tissues can generate an inflammatory milieu which further contributes to the dysfunction of aging muscle (Ilich et al., 2014). This suggests that the process of myosteatosis is multifaceted and bidirectional.

Myosteatosis exacerbates age‐related anabolic resistance (Breen & Phillips, 2011; Rivas et al., 2016) and affects the ability of the neighboring muscle tissue to manage oxidative stress (Hardin et al., 2008), ultimately impairing physical function. Myosteatosis (specifically increased IMAT and, to a lesser extent IMC) likely plays a central role (Addison, Marcus, et al., 2014; Inacio et al., 2014; Yoshida et al., 2012) by reducing muscle‐specific force per unit of cross‐sectional area (muscle quality) (Goodpaster et al., 2001; Hilton et al., 2008), thus contributing to the decline of physical function. Several studies demonstrate a connection between IMAT in the lower limbs and reduced mobility in functional measures such as gait speed (Beavers et al., 2013; Justice et al., 2018) and the short physical performance battery (SPPB) (Hilton et al., 2008; Tuttle et al., 2011, 2012). Myosteatosis has been further implicated in increased mortality risk, both in large population‐based studies (Reinders et al., 2016) and studies specific to older adults with chronic illness (Giani et al., 2022) or those currently hospitalized (Kang et al., 2022). However, this association has not been consistent across all studies, potentially due to inconsistencies between definitions of myosteatosis, the assessments used, and the muscles investigated (Correa‐de‐Araujo et al., 2020).

In this review, we aim to describe the broad mechanisms involved in the development of myosteatosis, summarize the clinical consequences of myosteatosis within skeletal muscle, and highlight current therapeutic treatment options aimed at ameliorating its negative impact on health and function.

### 1.1 Role of adipokines and inflammatory factors in the development of myosteatosis

**FIGURE 1:**
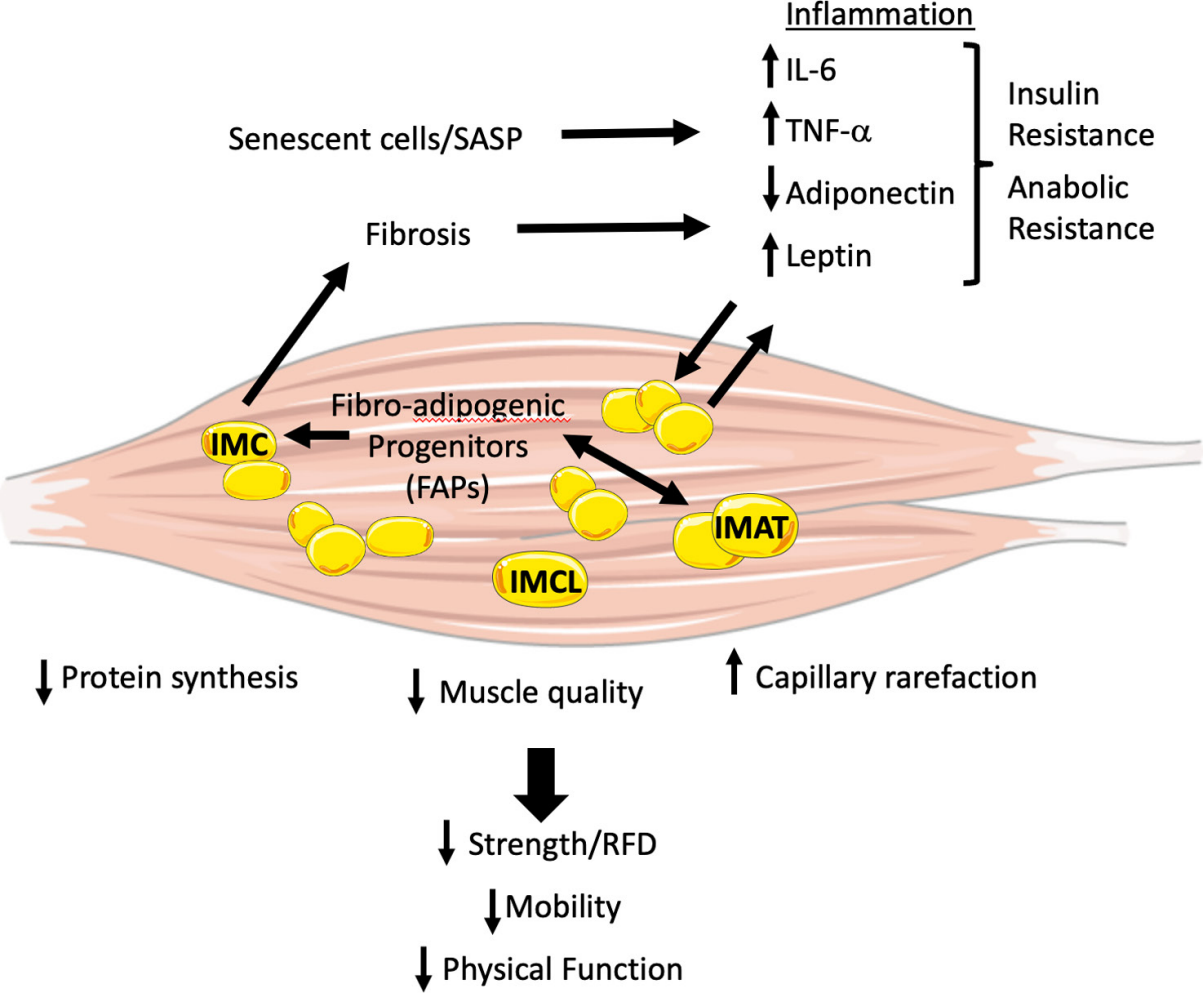
Myosteatosis, which includes intermuscular adipose tissue (IMAT), intramuscular adipose tissue (IMC), and intramyocellular lipid (IMCL) depots, is a complex condition that contributes to declines in the health and function of older adults. SASP, senescent‐associated secretory phenotype; RFD, rate of force development; IL‐6, interleukin‐6; TNF‐α, tumor necrosis factor‐α. Parts of the figure were drawn by using pictures from Servier Medical Art. Servier Medical Art by Servier is licensed under a Creative Commons Attribution 3.0 Unported License (https://creativecommons.org/licenses/by/3.0/).

Once thought to be an inert energy depot, adipose tissue is a dynamic endocrine tissue responsible for producing over 300 signaling and mediator molecules, collectively called adipokines (Lehr et al., 2012). Adipose depots are usually identified by location (e.g., subcutaneous, visceral, and intermuscular) and have the fundamental roles of storing lipids during rest and mobilizing lipids through lipolysis when sympathetically stimulated through fatty acid/triacylglycerol cycling (Sparks et al., 2021). Additionally, adipose tissue serves as a conversion site of androgens to estrogens outside of reproductive organs, playing a primary role in estrogen production as glandular production of estrogen declines later in life (Stout et al., 2017). While adipose tissue can confer metabolic benefits, it also can be metabolically detrimental in the presence of inflammatory conditions (Figure 1). Adipose tissue dysfunction typically arises when individual factors (such as genetics, diet, and exercise habits, as well as sex and the associated circulating hormones) negatively affect the metabolic health of these individual depots and disrupt their collective ability to maintain homeostasis—leading to metabolic disruption of neighboring tissues and adverse clinical outcomes (Sparks et al., 2021).

Though a more complete summary of the IMAT secretome has been published elsewhere (Kahn et al., 2022), the secretion of the adipokines leptin and adiponectin and the inflammatory factors IL‐6 and TNF‐α are important to highlight due to their integral roles in the metabolic homeostasis of adipose tissue (Ilich et al., 2014; Tagliaferri et al., 2015). Adiponectin typically promotes glucose and fatty acid homeostasis in skeletal muscle; thus, the impaired secretion of adiponectin contributes to the development of insulin resistance. Leptin can have both a positive and negative paracrine effect on nearby tissues such as bone and muscle (Harris, 2014; Tagliaferri et al., 2015). Leptin also has an autocrine effect on its leptin receptors, potentially contributing to leptin resistance and bone resorption (Harris, 2014; Tagliaferri et al., 2015). IL‐6 secreted by adipocytes, including those found in IMAT, can promote an inflammatory cascade, particularly when accompanied by other inflammatory factors such as TNF‐α (Stout et al., 2017), leading to chronic upregulation of inflammatory pathways and the blunting of anabolic and anticatabolic pathways. The infiltration of IMAT and the upregulation of these inflammatory pathways disrupt muscle homeostasis, eventually reducing the physical function of the muscle.

Due to its inflammatory secretome, IMAT is additionally associated with the accumulation of senescent cells, or cells in replicative arrest, which is typically attributed to the telomere shortening, DNA damage, and oxidative stress that occurs with aging (Stout et al., 2017). These senescent cells develop a highly inflammatory phenotype known as senescent‐associated secretory phenotype (SASP) and promote an inflammatory microenvironment through the secretion of inflammatory markers such as C‐reactive protein (CRP), interleukin‐1 beta (IL‐1β), IL‐6, and TNF‐α (Stout et al., 2017). Though IL‐6 is also a myokine and can be anti‐inflammatory when secreted during muscle contraction (Karstoft & Pedersen, 2016), the chronic secretion of IL‐6 from adipocytes in the presence of these other inflammatory markers leads to the dysregulation of glucose homeostasis and development of insulin resistance (Stout et al., 2017; Tagliaferri et al., 2015). The accumulation of lipids within the skeletal muscle has long been associated with insulin resistance, obesity, and glucose dysregulation (Goodpaster & Wolf, 2004). Thus, IMAT and the secretome of its associated adipokines contribute to the accumulation of chronic inflammation and the presence of metabolic dysfunction, creating a ripe microenvironment within the muscle for diminished muscle quality and the subsequent geriatric conditions of frailty and sarcopenia.

In addition to the role of adipokines in the promotion of metabolic dysfunction, inflammatory adipokines also differentiate local stem cells into adipocytes rather than myocytes (Renovato‐Martins et al., 2020; Zhu et al., 2015). This has been found to occur particularly in response to muscle fiber injury (Ki et al., 2021). Mesenchymal stem cells derived from adipocytes themselves, called preadipocytes or fibro‐adipogenic progenitors (FAPs), readily differentiate into adipocytes in the presence of muscle injury, thus increasing fatty deposition within and between fibers (Hamrick et al., 2016). The differentiation of FAPs into IMC or IMAT can also be accelerated in the presence of low‐grade inflammatory conditions such as aging, obesity, physical inactivity, leptin resistance, and loss of sex steroid hormones (Hamrick et al., 2016; Ilich et al., 2014; Pratesi et al., 2013). FAPs also begin to exhibit an inflammatory phenotype and activate an immune response (Stout et al., 2017). This is possibly due to activated adipose tissue macrophages shifting from the anti‐inflammatory M2 signaling pathway to the pro‐inflammatory M1 signaling pathway, thus activating reactive oxygen and nitrogen species production (Lumeng et al., 2007). Subsequently, insulin resistance, chronic systemic inflammation, and overall metabolic dysregulation are induced (Stout et al., 2017).

### 1.2 Clinical consequences of myosteatosis

#### 1.2.1 Muscle atrophy

Biological aging changes the composition and function of muscle tissue and related structures. The loss of muscle mass with aging is associated with decreases in the total muscle fiber number, a reduction in the diameter of the fibers, and muscle fiber denervation due to motor unit loss—which ultimately leads to muscle atrophy (Ferrucci et al., 2014). While reinnervation mechanisms are impaired in older adults, some reinnervation does occur, resulting in fiber type transitions of type 2/fast twitch fibers to that of Type 1 fibers, which reduces fast twitch muscle function. Throughout this process, there is a concomitant increase in IMC and IMCL infiltration into the existing fibers, further reducing functional capabilities (Bellanti et al., 2021; Ferrucci et al., 2014). As noted earlier, this is partly due to the differentiation of nearby mesenchymal stem cells into adipocytes rather than myocytes, but can also be due to disuse, sex steroid hormone deficiency with aging, or leptin deficiency/resistance (Hamrick et al., 2016). Muscle fiber orientation may be subsequently altered by this infiltration of lipids between the muscle fibers, resulting in a decreased capacity for force production which has strong clinical implications for the function of the muscle (Marcus et al., 2012).

As previously discussed, myosteatosis exacerbates and contributes to local inflammation, reducing insulin sensitivity (Correa‐de‐Araujo et al., 2020; Visser, Pahor, et al., 2002). This has clinical consequences, compounding the age‐related attenuation of muscle protein synthesis and decreased leucine sensitivity that many older adults are already experiencing (Cuthbertson et al., 2005). Contemporary perspectives consider reduced muscle protein synthesis to be due to an amino acid imbalance and decreased sensitivity to anabolic stimuli, such as insufficient or low‐quality dietary protein intake and lack of physical activity (Breen & Phillips, 2011; Ferrucci et al., 2014). The amino acid imbalance is accompanied by the age‐related reduction in anabolic hormone response, such as reduced growth hormone (GH), insulin‐like growth factor 1 (IGF‐1), and testosterone, which blunts the anabolic activity of the mammalian target of rapamycin (mTOR) pathway and other anticatabolic pathways. Myosteatosis may further contribute to anabolic resistance by upregulating the ubiquitin‐proteasome pathway, stimulated by the presence of inflammatory cytokines and thus reducing protein phosphorylation in the mTOR pathway essential for muscle protein synthesis (Breen & Phillips, 2011; Rivas et al., 2016).

Rivas et al. demonstrated reduced anabolic signaling following resistance exercise in older adults associated with altered ceramide content secondary to increased intramyocellular lipids. This study further demonstrates an inability of aged skeletal muscle to phosphorylate and inhibit FOXO1 (forkhead box transcription factor O1), an essential regulator of the ubiquitin‐proteasome pathway, following an acute bout, which may explain in part the reduced response to exercise seen in older adults (Rivas et al., 2012).

#### 1.2.2 Capillary rarefaction

The reduction in muscle function due to IMAT infiltration is also potentially affected by changes in capillary density (Addison et al., 2020; Prior et al., 2016). While it has been less studied, theoretically changes in capillarization may also be related to the accumulation of IMAT within a muscle. Diminished skeletal muscle capillarization can limit oxygen, amino acid, hormone, and nutrient delivery, which in turn contributes to age‐related decreases in muscle mass, function, and metabolism (Landers‐Ramos & Prior, 2018). Increased levels of IMAT are also frequently found in populations known to have decreased capillarization of skeletal muscle, such as those with type 2 diabetes (Landers‐Ramos & Prior, 2018). Deposition of IMAT around capillaries in the muscle may contribute by further restricting substrate and anabolic stimuli delivery to the muscle (Biltz et al., 2020; Prior et al., 2016). While it has not yet been extensively studied, a cross‐sectional study by Addison et al. previously found that increased levels of capillarization were related to increased levels of IMAT within a muscle (Addison et al., 2020). While there were no clear explanations for this finding, they theorized that the paradoxical results may be related to higher levels of IMAT promoting a pro‐inflammatory environment, resulting in an expansion of IMAT and ultimately muscle fibrosis. Further work is necessary to explore the relationships between changes in capillarization, changes in IMAT, and muscle dysfunction.

#### 1.2.3 Neuromuscular activation and impact on mobility

While myosteatosis has been independently associated with reduced maximal torque production in longitudinal studies (Delmonico et al., 2009; Goodpaster et al., 2001), the decreased force production may be independent of atrophy as IMAT is thought to inhibit neuromuscular activation resulting in impaired contractions (Biltz et al., 2020; Yoshida et al., 2012). Although a small study, Yoshida et al. observed an impaired central activation ratio in the knee extensors of older adults with high levels of IMAT (Yoshida et al., 2012). Increased secretion of pro‐inflammatory cytokines such as TNF‐α associated with IMAT may contribute to further reductions in force capacity even in the absence of muscle atrophy (Hardin et al., 2008). Evidence shows that IMAT induces a greater inflammatory response than its subcutaneous and visceral counterparts, suggesting that IMAT is especially influential in chronic inflammation and has a strong paracrine influence on the maintenance of homeostasis in neighboring muscle (Hardin et al., 2008; Kahn et al., 2022). The ability to generate strength and power, especially from the muscles of the lower extremity, is integral for maintaining mobility and function throughout aging. Large epidemiologic studies have described an association between IMAT, current mobility deficits, and increased risk for future loss of mobility (Addison, Marcus, et al., 2014; Marcus et al., 2012; Visser et al., 2005; Visser, Kritchevsky, et al., 2002). This is especially true with its presence in the muscles of posture and locomotion, particularly the hip abductors. The proximal muscles of the hip, namely the gluteal muscles, may be particularly vulnerable to fatty infiltration and are important for balance function in older adults (Inacio et al., 2014). Self‐reported fallers had higher levels of lower extremity myosteatosis across all muscle but most significantly in the proximal muscles of the hip (Inacio et al., 2014). Individuals classed as “impaired steppers” (i.e., those who cannot recover quickly from balance perturbations and are therefore more likely to experience falls) show higher degrees of myosteatosis in the hip abductors and lower hip abduction torque (Addison et al., 2017). Interestingly, myosteatosis also varies in severity and its impact on functional outcomes depending on the muscle group measured. For example, IMAT content of the gluteal muscles was associated with reductions in overall balance and increased gait variability (an established predictor of general mobility function), but IMAT in the thigh muscles did not demonstrate this association (Addison, Young, et al., 2014). These findings point to the importance of muscle quality and its role in maintaining physical function in older adults and highlight the need for assessment of specific muscle groups.

#### 1.2.4 Changes in muscle stiffness and function

The presence of myosteatosis is also attributed to increasing muscle stiffness with age (Marcucci & Reggiani, 2020; Xu et al., 2021). As mentioned previously, Addison et al. demonstrated that increased IMAT is associated with an increase in capillarization, potentially supporting the development of fibrosis in the extracellular matrix (ECM) (Addison et al., 2020). This may partially explain why myosteatosis is associated with increased muscle stiffness. Muscle stiffness can be affected by sarcomere length, the number and type of collagen present within the muscle, and the homeostasis of the extracellular matrix (ECM) (Lieber & Binder‐Markey, 2021). In instances of aging and chronic disease, the enzymatic and non‐enzymatic cross‐linking of collagen structures within the ECM contributes to increases in passive muscle stiffness (Lieber & Binder‐Markey, 2021; Xu et al., 2021). The lack of cellular homeostasis and subsequent levels of chronic inflammation within aging muscle sustains the progressively detrimental positive feedback loop of FAPs (Loomis & Smith, 2023). FAPs, which typically would increase adipocyte differentiation and support muscle regeneration, instead continue to be activated in a positive feedback loop in the presence of increasing fibrosis and inflammatory cytokines such as transforming growth factor‐beta (TGF‐β) (Loomis & Smith, 2023). Thus, in the presence of chronic inflammation, FAPs are unable to follow their traditional regulating apoptotic pathway and instead activate into myofibroblasts and adipocytes (Loomis & Smith, 2023). When FAPs are dysregulated, adipose tissue infiltrates the muscle and ECM disruption occurs, thus increasing fibrosis and fatty infiltration through the disruption of fiber alignment and replacement of contractile tissue (Lieber & Binder‐Markey, 2021; Loomis & Smith, 2023; Xu et al., 2021). Additionally, because FAPs are no longer transiently stimulated during muscular injury, they lose their role in stimulating myocellular repair, become more pathologic in nature, and recovery from muscle microinjury is blunted (Loomis & Smith, 2023). IMC infiltration becomes more likely as fatty deposits replace damaged muscle fibers and adipose tissue dysfunction propagates metabolic dysfunction (Biltz et al., 2020).

Currently, there are limited clinical methods to evaluate muscle stiffness. In research studies at the microscopic level, fiber dissection of both animal models and human muscle biopsies are common, enabling the assessment of the presence and density of collagen isoforms (Lieber & Binder‐Markey, 2021) and the degree of collagen cross‐linking in the ECM (Lieber & Binder‐Markey, 2021). At the macroscopic level, musculoskeletal ultrasound utilizing shear wave elastography has been used to evaluate passive mechanics at the whole muscle level (Alfuraih et al., 2018; Lieber & Binder‐Markey, 2021; Xu et al., 2021). Yoshiko et al found that passive gastrocnemius stiffness was positively associated with IMAT content in young individuals (Yoshiko et al., 2023). However, the implications of these findings are limited as stiffness measurements are restricted to the probe region and stiffness is not uniform across an entire muscle (Lieber & Binder‐Markey, 2021). Thus, an active area of research is to develop clinical methods that can be utilized to assess muscle stiffness. Further work is necessary to develop clinically relevant and reliable assessment methods that can be utilized by clinicians.

### 1.3 Potential therapeutic treatment interventions

#### 1.3.1 Pharmacological treatment targets

Although complete prevention of myosteatosis is unrealistic, therapeutic targets aimed at slowing its accumulation have been a central focus of research (Correa‐de‐Araujo et al., 2020). Xie and colleagues recently summarized several mouse models that have been leveraged to study myosteatosis and sarcopenia, helping to identifying potential therapeutic targets for this condition (Xie et al., 2021). For example, adiponectin is a means of regulating lipid metabolism and signaling when promoted, and has been a target of myosteatosis pharmacologic interventions (Diep Nguyen, 2020). In mice with obesity, an adiponectin receptor agonist improved mitochondrial function and lipid oxidation thereby reversing age‐related IMC accumulation. Additionally, functional improvements in endurance were observed (Selvais et al., 2023). While adiponectin receptor agonists have not yet been tested in humans, harnessing this pathway is a future area of research.

Another pharmacologic target in the treatment of myosteatosis is the inhibition of myostatin. Myostatin inhibits muscle protein synthesis and is more strongly expressed in adults with higher levels of muscle fatty infiltration (Becker et al., 2015; Zoico et al., 2013). Mice with a myostatin deletion show increased lean mass, decreased adipose tissue mass, and improved metabolic functioning (Guo et al., 2009). Based on this concept, Becker et al. completed a Phase 2 clinical trial of LY2495655, a myostatin monoclonal antibody, in older adults with a history of falls. Patients receiving LY2495655 showed increased appendicular lean body mass and a trend toward improvements in functional measures of muscle power (Becker et al., 2015). To date, no Phase 3 clinical trials of LY295655 have been reported.

Aging adults with obesity and/or type 2 diabetes are more likely to have ectopic fatty deposits (Sparks et al., 2021). This association is bidirectional, with IMAT impacting glucose uptake and hyperglycemia‐inducing fat storage within the muscle and other tissues (Miljkovic & Zmuda, 2010). Therefore, sodium‐glucose cotransporter 2 (SGLT‐2) inhibitors and glucagon‐like peptide‐1 (GLP‐1) receptor agonists which improve glucose control and have been shown to induce fat loss have been posited as potential treatment options for myosteatosis (Ma et al., 2023). SGLT‐2 inhibitors work by inhibiting proximal tubular glucose reabsorption, thus increasing the amount of urinary glucose excretion (Pereira & Eriksson, 2019), while GLP‐1 receptor agonists augment insulin secretion, suppress glucagon secretion, and slow gastric emptying in response to meal‐related glucose excursions (Nauck et al., 2021). Meta‐analyses have found patients using SGLT‐2 inhibitors were able to preserve lean body mass while decreasing fat mass (Pan et al., 2022), while those using GLP‐1 receptor agonists experienced 20%–50% reductions in lean body mass with weight loss (Sargeant et al., 2019). No existing studies have directly examined effects on IMAT; however, this research suggests that SGLT‐2 inhibitors may improve muscle quality with weight loss while improving glucose control. Thus, the effect of these medications on IMAT specifically should be a targeted future area of research.

#### 1.3.2 Nutritional and exercise therapeutic interventions

Nutritional interventions with the goal of weight loss have been explored for the management and treatment of myosteatosis. Caloric restriction interventions (prescribed as 16%–20% caloric deficit per day) lasting 16–20 weeks cause weight loss including loss of muscle mass, however, favorable muscle composition changes also occur with associated declines in low‐density, lipid‐rich, muscle (Brinkley et al., 2011; Correa‐de‐Araujo et al., 2020; Murphy et al., 2012; Shea et al., 2011). Furthermore, up to a 40% reduction in soleus IMCL content was observed in overweight and obese adults with type 2 diabetes following a paleolithic diet for 12 weeks (Otten et al., 2018). Shea et al. found females lost more lean muscle mass than males with a weight loss intervention even though comparable changes in body weight were observed in both sexes, highlighting the need for sex‐specific considerations with myosteatosis nutritional intervention (Shea et al., 2011). Regardless of sex, those experiencing reductions in thigh IMAT with weight loss demonstrate improvements in physical functioning (Santanasto et al., 2011, 2015).

Improving physical functioning in older adults has also been proposed to improve the metabolic health of the muscle and thus reduce the presence or severity of myosteatosis (Addison, Marcus, et al., 2014). Up to 15% of the variance in mobility performance in older adults can be explained by IMAT volume alone (Marcus et al., 2012), and fatty infiltration in muscle can independently predict which healthy older adults will develop mobility limitation as they age (Visser et al., 2005). Higher IMAT volume is associated with shorter 6‐minute walk distance, slower stair negotiation speed, poorer global physical performance, and lower physical activity levels in older adults (Marcus et al., 2012; Tuttle et al., 2011, 2012). Mechanical stimulation of muscle fibers reduces intramuscular fat accumulation; however, existing fat depositions can impair contractions (Rubin et al., 2007; Yoshida et al., 2012). Therefore, physical activity and exercise interventions implementing different modes of activity are recommended for the treatment of older adults with myosteatosis.

A meta‐analysis of exercise interventions for those with myosteatosis found exercise interventions, on average, reduced lipid infiltration and increased skeletal muscle attenuation coefficient (a marker of muscle density and decreased IMC, as determined by computed tomography) in older adults (Ramírez‐Vélez et al., 2021). In particular, aerobic exercise and concurrent aerobic and strength training (Otten et al., 2018) were strongly associated with decreased lipid infiltration. The strongest evidence for increasing muscle attenuation was during a concurrent aerobic and strength training program in older women with type 2 diabetes where an effect size of 2.3 was observed (Cuff et al., 2003). Prior et al. found older males also experience increased muscle attenuation when participating in aerobic exercise, especially when accompanied by weight loss (Prior et al., 2007). However, improvements in muscle quality may only be attainable through exercise in those with initially low levels of myosteatosis. (Marcus et al., 2013). Because the decline in muscle quality is often multifactorial, it is possible that age and the presence of co‐existing chronic conditions in addition to myosteatosis may blunt anabolic training responses. Overall, this area of research is limited by the small sample sizes with group sizes of <30 often observed. Future studies should focus on larger sample sizes to establish population validity, and multi‐modal rehabilitation approaches addressing muscle‐sparing fat loss and exercise may be warranted for treatment of myosteatosis. Future advances in pharmacologic interventions may also aid nutritional and activity‐based interventions in more efficiently combating intramuscular fatty deposition to improve function and muscle quality in aging adults with obesity vulnerable to decline.

## 2 CONCLUSIONS

Myosteatosis heavily exacerbates the negative physiological changes that occur with aging, creating complicated pathologies that are difficult to disentangle and rescue. Muscle and adipose tissue dysfunction both promote an inflammatory, catabolic state—encouraging the infiltration of adipose tissue and resulting in the loss of force production (Correa‐de‐Araujo et al., 2020; Xu et al., 2021). Though age‐related cellular senescence may precede or coincide with many of the above changes, it ultimately promotes a more pathological cellular environment and contributes to an overall decline in muscle quality (Correa‐de‐Araujo et al., 2017). Though there is promising research aimed at various therapeutic targets to mitigate myosteatosis, success has been limited thus far, and many options are not potent enough to completely rescue the metabolic and physical consequences of myosteatosis. Future research is encouraged for the development of reliable assessment methods for myosteatosis, as well as the continued exploration of pharmacological, nutritional, and exercise‐related interventions that may lead to the success in attenuating myosteatosis and its clinical consequences within the aging population.

## AUTHOR CONTRIBUTIONS

K.D. and O.A. outlined the content of this manuscript. All authors drafted the manuscript and R. L‐R. developed the figures and graphical abstract. All authors contributed manuscript edits and approved the final version of this manuscript.

## FUNDING INFORMATION

The National Institute on Aging (NIA) Claude D. Pepper Older Americans Independence Center P30‐AG028747. This manuscript was prepared using protected time as part of the Advanced Fellowship in Geriatrics, supported by US Department of Veterans Affairs Office of Academic Affiliations, the Veterans Affairs Maryland Health Care System, and the Department of Veterans Affairs Baltimore Geriatric Research, Education, and Clinical Center (GRECC). The views expressed in this article are those of the authors and do not necessarily represent the position or policy of the U.S. Department of Veterans Affairs or the United States Government.

## 3 ETHICS STATEMENT

The authors do not have any conflicts of interest to disclose, and confirm that this manuscript is not published elsewhere.
